# The role of neutrophil extracellular traps in cancer metastasis

**DOI:** 10.1002/ctm2.126

**Published:** 2020-09-22

**Authors:** William Wang, Jiayang Zhang, Nanan Zheng, Li Li, Xiangdong Wang, Yiming Zeng

**Affiliations:** ^1^ Zhongshan Hospital Institute of Clinical Science, Fudan University Shanghai Medical College Center for Tumor Diagnosis and Therapy, Jinshan Hospital Fudan University Shanghai China; ^2^ Key Laboratory of Carcinogenesis and Translational Research (Ministry of Education/Beijing) Department of Breast Oncology Peking University Cancer Hospital & Institute Beijing China; ^3^ Department of Pulmonary and Critical Care Medicine Clinical Center for Molecular Diagnosis and Therapy The Second Affiliated Hospital of Fujian Medical University Quanzhou Fujian Province China; ^4^ Center for Clinical Single Cell Biomedicine, Henan Provincial People's Hospital People's Hospital of Zhengzhou University Zhengzhou Henan China

Extracellular traps are web‐like structures and consist of chromatin DNA filaments, histones, and granule proteins. These are released together with inflammatory mediators and pathogens after the stimulation of inflammatory cells, for example, neutrophils, monocytes, eosinophils, mast cells, and macrophages. Extracellular trap production and activation are immune defense mechanisms that contribute to the development of inflammation, immune hyperresponsiveness, hemodynamics alterations, endothelial barrier function, and induction of tumor microenvironment heterogeneity. Neutrophil extracellular traps (NETs) are released by neutrophils to capture and eliminate pathogens during DNA expulsion (NETosis), a regulated form of neutrophil cell death. The production of NETs and NETosis formations are considered as an evolutionary process, of which disorder and dysregulation can cause a large number of diseases. Preclinical studies demonstrated that NETs and NETosis could play critical roles in the pathogenesis of inflammation, infection, thrombosis, tissue injury, organ dysfunction, and cancer metastasis. NETs mainly contain neutrophil elastase, myeloperoxidase, cathepsin G, proteinase 3, lactoferrin, gelatinase, lysozyme C, calprotectin, neutrophil defensins, and cathelicidins. The aim of this editorial is to provide an update on the latest investigations regarding the interaction between NETs and cancer cells, the potential of NETs and NETs‐associated elements in the identification and development of cancer‐specific biomarkers and targets, and the value of clinical translation from NETs biology.

NETs regulate cancer cell activities and metabolisms, while cancer‐associated extracellular vesicles modulate neutrophil behavior and NET extrusion. The complexity of molecular mechanism by which NETs and NETosis contribute to cancer cell biology have exceeded our expectations. For example, cancer‐associated inflammation can generate intra‐ and extracellular environments for neutrophil activation and can produce reactive oxygen species via NETs/NETosis release resulting in systemic oxidative stress. Cancer cells can act as the primary receptor, interacting directly with cytokines/chemokines from other cells, and simultaneously as the secondary producer of those factors to chemoattract the recruitment of inflammatory cells into the microenvironment. Cancer cells are decisive factors in cancer microenvironment formation and composition, inflammatory cell recruitment and activation, and NETs/NETosis production. The number of enzymes (eg, DNAse, peptidylarginine deaminase, neutrophil elastase, and myeloperoxidase), receptors (eg, Toll‐like receptor 4, 9; receptor for advanced glycation end products; integrins; lectins), intracellular mediators (eg, high mobility group box 1), and hypoxia are critical factors in the production of NETs and are responsible for cancer cell adhesion, proliferation, migration, and invasion.^1^

The production of NETs and NETosis formations seems to be regulated by either radical‐dependent or independent pathways. Brinkmann systemically overviewed the understanding of NETs and summarized the inducers for NETs/NETosis (eg, bacteria or bacterial components, fungi, protozoa, viruses, activated platelets, complement‐derived peptides, autoantibodies, IL‐8, hydrogen peroxide, urate crystals, cigarette smoke, and ionophores).^2^ Histone citrullination is a characteristic of NETosis that can be generated by peptidylarginine deaminase‐catalyzed hypercitrullination in the histones H3, H2A, and H4These enzymes are involved in altering chromatin structure and decondensation by binding with DNA, participating in the oxidative reaction, relocating between cytoplasm and the nucleus, and decorating the DNA backbone of NET fibers. NETs production and release is orchestrated by altered chromatin, disordered cell morphology, the ruptured nuclear envelope, and plasma membrane and is driven by the major physical force known as entropic chromatin swelling that is detected in real‐time on the single‐cell level using fluorescence and atomic force microscopy.^3^ Neubert et al defined the NETosis process as “a clear point of no return” with three distinct phases, that is, cell activation and initiation of chromatin expansion with intact lobular structures of the nucleus, maximum chromatin expansion near the cellular membrane, rupturing of the cell membrane, and the release of NETs into extracellular space.^3^ The process of NETosis could be altered if inflammation is induced by lipopolysaccharides or if there are alterations within ion channels.

Cancer cells can interact directly and indirectly with neutrophils to induce the production of NETs through the production of extracellular vesicles and proinflammatory mediators (eg, cytokines, chemokines, and tissue factors). Such interactions provoke platelets and endothelia to initiate the inflammatory cascade and to promote the release of NETosis and NETs via surface antigens (eg, P‐selectin/P‐selectin glycoprotein ligand 1). Intra‐ and intercellular molecular communications during multicellular interactions create new distant microenvironments that act as a pre‐metastatic niche to attract the recruitment of primary cancer cells and to form multiple metastases. It is proposed that NETs play multiple critical roles in the promotion of cancer metastasis after surgery through the suppression of systemic immune functions, onset of tissue damage‐induced inflammation, activation of damage‐associated molecular patterns, and hyperresponsiveness of tumor‐associated macrophages, myeloid‐derived suppressor cells, or/and regulatory T cells.^4^

Preclinical and clinical observations evidenced the activation and release of NETs and NETosis in patient samples, although the roles as primary or secondary factors in the development of diseases remain unclear. NETs can directly contribute to the development of brain parenchyma pathology, different from neutrophils per se that are restricted by the brain‐blood barrier. NETs were characterized by citrullinated histone H3 and observed in patients with thrombosis‐associated ischemic stroke, especially higher in older thrombi. NETs appeared in the cerebrospinal fluid of patients with pneumococcal meningitis, rather than in other forms of meningitis caused by viruses, Borrelia, and subarachnoid hemorrhage.^5^ This indicates that the production of NETs may be pathogen‐specific and infection‐specific as well as contribute to the development of the disease, which was further confirmed by preclinical evidence that NETs appeared in the cerebrospinal fluid of animal meningitis induced by the clinical strain of pneumococci. NETs were found to be in the outer layers of thrombi in patients with acute ischemic stroke and was associated with endovascular therapy procedure length and the number of device passes, rather than the clinical phenomes of patients.^6^ NETs in the brain can exacerbate the process of tissue injury by activating intracellular mediators (eg, high mobility group box 1).

Multiple factors are involved in the process of cancer‐associated NETs and NETosis, and various biomarkers have been proposed to monitor and predict the progression, response to drugs, and prognosis of cancers. For example, elevated plasma levels of citrullinated histone H3 were considered as a marker to show the severity of NETs release associated with advanced cancer and metastasis and correlated with the neutrophil activation markers neutrophil, elastase, and myeloperoxidase as well as with poor clinical prognosis.^7^ In addition, regulators involved in the process of chromatin expansion can be candidates for determining the initiation and maximum of chromatin expansion the at the early stage of NETs production (Figure 1), for example, DNA damage‐associated poly(ADP‐ribose) polymerase 1 (PARP1), chromatin remodeler CHD2, citrullinated histone H3 isoforms, polycomb repressive complexes, nuclear F‐actin, zinc finger protein CCCTC‐binding factor, histone methyltransferase NSD2, cyclin‐dependent kinases, and DNase 1‐like protein 3. At the final stage of NETosis, neutrophil elastase, myeloperoxidase, cathepsin G, proteinase 3, lactoferrin, gelatinase, lysozyme C, calprotectin, neutrophil defensins, and cathelicidins are released with NETs resulting in alterations within the local and distant microenvironments and the formation of metastatic niches. NETs are also considered as a predictive biomarker for multi‐cancers. However, it is questioned whether NETs and NETs‐associated factors have the specificity for disease categories, phenomes, severities, stages, durations, metastasis, and responses to drugs as criteria for disease‐specific biomarkers.^8^, ^9^, ^10^, ^11^, ^12^, ^13^, ^14^ Cancer‐associated factors appear during the development of new NETs‐targeted therapies (Figure 1) and is evidenced by preclinical cancer models and clinical cancer patients. It is proposed that anti‐DAMPs, anti‐postoperative inflammation, inflammatory/pyroptosis signals, immunotherapy with surgery, antiangiogenesis, and targeted therapies for neutrophils, macrophages, MDSCs, and Tregs could be a new therapeutic strategy to reduce cellular immunity impairment after surgery.^3^ On basis of the biochemical properties of those therapeutic targets, it is possible to identify and develop NETs‐target therapies for cancer metastasis, although the specificity and efficacy need to be furthermore defined.

**FIGURE 1:**
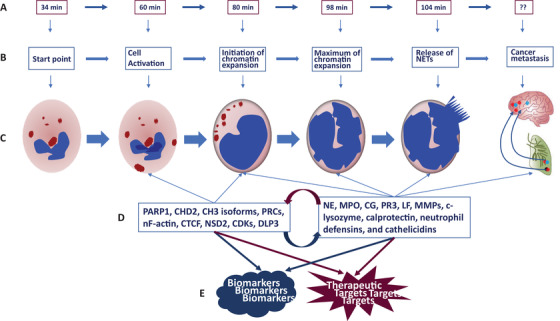
The process of NETosis formation and NETs release and the potential of NETs‐associated biomarkers and targets. The time of NETs release was reported^3^, while the time during which NETs‐associated factors interact with other cells remains unclear (A). The process is divided into different phases (B), during which the cell is activated to initiate the process of chromatin expansion with intact lobular structures of the nucleus, maximum of chromatin expansion near the cellular membrane till the full round size, and rupture of the cell membrane and the release of NETs into the extracellular space (C). The number of NETs‐associated inducers, regulators, and enzymes contribute to the different phases (D) and may be translated into diagnostic biomarkers and therapeutic targets (E)

In conclusion, the overproduction of NETs‐associated regulators and factors and the creation of new metastatic niches that attract and recruit cancer cells highlights the importance of NETosis formation and NETs release. The number of NETs inducers, regulators, and products are considered as diagnostic biomarkers and therapeutic targets for clinical translation. NETs‐target inhibition will provide novel alternative therapeutic strategies for cancer metastasis.

## CONFLICT OF INTEREST

The authors have declared no conflict of interest.

## References

[1] Snoderly HT , Boone BA , Bennewitz MF . Neutrophil extracellular traps in breast cancer and beyond: current perspectives on NET stimuli, thrombosis and metastasis, and clinical utility for diagnosis and treatment. Breast Cancer Res. 2019;21(1):145. 31852512 10.1186/s13058-019-1237-6 PMC6921561

[2] Brinkmann V . Neutrophil extracellular traps in the second decade. J Innate Immun. 2018;10(5‐6):414‐421. 29909412 10.1159/000489829 PMC6784051

[3] Neubert E , Meyer D , Rocca F , et al. Chromatin swelling drives neutrophil extracellular trap release. Nat Commun. 2018;9(1):3767. 30218080 10.1038/s41467-018-06263-5 PMC6138659

[4] Tang F , Tie Y , Tu C , Wei X . Surgical trauma‐induced immunosuppression in cancer: recent advances and the potential therapies. Clin Transl Med. 2020 Jan;10(1):199‐223. 32508035 10.1002/ctm2.24 PMC7240866

[5] Mohanty T , Fisher J , Bakochi A , et al. Neutrophil extracellular traps in the central nervous system hinder bacterial clearance during pneumococcal meningitis. Nat Commun. 2019;10(1):1667. 30971685 10.1038/s41467-019-09040-0 PMC6458182

[6] Ducroux C , Di Meglio L , Loyau S , et al. Thrombus neutrophil extracellular traps content impair tpa‐induced thrombolysis in acute ischemic stroke. Stroke. 2018;49(3):754‐757. 29438080 10.1161/STROKEAHA.117.019896

[7] Thålin C , Lundström S , Seignez C , et al. Citrullinated histone H3 as a novel prognostic blood marker in patients with advanced cancer. PLoS One. 2018;13(1):e0191231. 29324871 10.1371/journal.pone.0191231 PMC5764486

[8] Chirshev E , Oberg KC , Ioffe YJ , Unternaehrer JJ . Let‐7 as biomarker, prognostic indicator, and therapy for precision medicine in cancer. Clin Transl Med. 2019;8(1):24. 31468250 10.1186/s40169-019-0240-y PMC6715759

[9] Qi X , Yu C , Wang Y , Lin Y , Shen B . Network vulnerability‐based and knowledge‐guided identification of microRNA biomarkers indicating platinum resistance in high‐grade serous ovarian cancer. Clin Transl Med. 2019;8(1):28. 31664600 10.1186/s40169-019-0245-6 PMC6820656

[10] Mirtavoos‐Mahyari H , Ghafouri‐Fard S , Khosravi A , et al. Circulating free DNA concentration as a marker of disease recurrence and metastatic potential in lung cancer. Clin Transl Med. 2019;8(1):14. 31001798 10.1186/s40169-019-0229-6 PMC6473013

[11] Ansari D , Torén W , Zhou Q , Hu D , Andersson R . Proteomic and genomic profiling of pancreatic cancer. Cell Biol Toxicol. 2019;35(4):333‐343. 30771135 10.1007/s10565-019-09465-9 PMC6757097

[12] Gil J , Betancourt LH , Pla I , et al. Clinical protein science in translational medicine targeting malignant melanoma. Cell Biol Toxicol. 2019;35(4):293‐332. 30900145 10.1007/s10565-019-09468-6 PMC6757020

[13] Qiao T , Wang X . A new light of proteomics in cell biology and toxicology. Cell Biol Toxicol. 2019;35(4):289‐291. 31428956 10.1007/s10565-019-09492-6

[14] Rivera‐Franco MM , Leon‐Rodriguez E , Torres‐Ruiz JJ , Gómez‐Martín D , Angles‐Cano E , de la Luz Sevilla‐González M . Neutrophil extracellular traps associate with clinical stages in breast cancer. Pathol Oncol Res. 2020;26:1781‐1785. 31656990 10.1007/s12253-019-00763-5

